# Use of Social Media in the Assessment of Relative Effectiveness: Explorative Review With Examples From Oncology

**DOI:** 10.2196/cancer.7952

**Published:** 2018-06-08

**Authors:** Rachel RJ Kalf, Amr Makady, Renske MT ten Ham, Kim Meijboom, Wim G Goettsch, IMI-GetReal Workpackage 1

**Affiliations:** ^1^ National Health Care Institute Diemen Netherlands; ^2^ Department of Pharmacoepidemiology and Clinical Pharmacology Utrecht University Utrecht Netherlands; ^3^ Department of Health Sciences VU University Amsterdam Amsterdam Netherlands

**Keywords:** social media, relative effectiveness, real-world data, patient reported outcomes

## Abstract

**Background:**

An element of health technology assessment constitutes assessing the clinical effectiveness of drugs, generally called relative effectiveness assessment. Little real-world evidence is available directly after market access, therefore randomized controlled trials are used to obtain information for relative effectiveness assessment. However, there is growing interest in using real-world data for relative effectiveness assessment. Social media may provide a source of real-world data.

**Objective:**

We assessed the extent to which social media-generated health data has provided insights for relative effectiveness assessment.

**Methods:**

An explorative literature review was conducted following the Preferred Reporting Items for Systematic Reviews and Meta-Analyses guidelines to identify examples in oncology where health data were collected using social media. Scientific and grey literature published between January 2010 and June 2016 was identified by four reviewers, who independently screened studies for eligibility and extracted data. A descriptive qualitative analysis was performed.

**Results:**

Of 1032 articles identified, eight were included: four articles identified adverse events in response to cancer treatment, three articles disseminated quality of life surveys, and one study assessed the occurrence of disease-specific symptoms. Several strengths of social media-generated health data were highlighted in the articles, such as efficient collection of patient experiences and recruiting patients with rare diseases. Conversely, limitations included validation of authenticity and presence of information and selection bias.

**Conclusions:**

Social media may provide a potential source of real-world data for relative effectiveness assessment, particularly on aspects such as adverse events, symptom occurrence, quality of life, and adherence behavior. This potential has not yet been fully realized and the degree of usefulness for relative effectiveness assessment should be further explored.

## Introduction

Within the context of rising health care costs, limited budgets, and the onslaught of innovative yet expensive medications, the value of health technology assessment (HTA) for decision-makers, regulators, pharmaceutical companies and patients is becoming increasingly important. HTA is defined as “the systematic evaluation of the properties and effects of a health technology” [1]. Health technologies are defined as “interventions developed to prevent, diagnose or treat medical conditions, promote health, provide rehabilitation, or organize health care delivery” [2]. An important element of HTA is relative effectiveness, ie, the extent to which an intervention – provided under routine clinical conditions – does more good than harm in comparison to one or more alternatives [1]. Traditionally, a relative effectiveness assessment (REA) conducted directly after-market authorization of a new drug is extrapolated using health outcomes (eg, mortality) obtained from randomized controlled trials (RCTs), which are often considered the gold standard for this type of analysis. However, the tightly-controlled conditions and highly selective patient groups within RCTs may result in findings that are not generalizable to routine clinical settings where patients are more heterogeneous. In routine practice, pregnant women, children, elderly people and patients with comorbidities may eventually receive the new drugs examined in RCTs, while these patient populations are generally excluded from such RCTs. Therefore, researchers may additionally resort to real-world data (RWD) as a supplementary source of evidence to assess relative effectiveness. Real-world data can be defined as “an umbrella term for data regarding the effects of health interventions that are not collected in the context of conventional randomized controlled trials” [1]. Patient registries and electronic health records are established examples of RWD sources, but another potential source of RWD may be social media.

Social media are often used by patients as a source to search for information on their health conditions, share their experiences and find social support [3,4]. For example, many patients use Twitter to stay up to date with the latest health care developments and increase their knowledge on their disease, while Facebook is more often used for social support and exchanging experiences [3]. Social media users who have a chronic condition are more likely to use the internet for such purposes than are healthy social media users [5]. By assessing the content viewed, generated and exchanged by patients through social media, a considerable amount of information on patient perspectives and experiences can be gathered. Although social media have been used for different aspects of research, such as patient recruitment [6-8], dissemination of interventions [9,10] and education [11], little is known about its contribution to REA.

In 2008 a study showed that blogs could be used to collect patient experiences regarding diabetes and diabetes management to provide information for HTA by enhancing the evidence available in published literature [12]. More recently, several pharmaceutical companies have begun to make use of social media to gain insight into patient perspectives on adverse events (AEs) [13,14] and to assess their switching behaviors [15]. Similarly, the Association of the British Pharmaceutical Industry (ABPI) has published guidelines on best practices for the monitoring and management of AEs through such sources [16]. Moreover, the Food and Drug Administration (FDA) is increasingly focusing on the use of health data from social media by collaborating with PatientsLikeMe; a platform where patients can share their health data online to gain insight into patient perspectives on adverse events [17,18]. Considering these initiatives, it may become possible for health data reported by patients on social media to contribute to the REA of new therapies.

The aim of this article is to assess the extent to which health data generated from social media have provided insights for REA. We conducted an explorative review to identify examples in oncology where health data were collected using social media. Oncology was chosen due to the considerable number of innovative drugs being developed at a rapid pace in this area. For example, the European Medicines Agency reported in 2015 that one-third of the medicines with a new active substance recommended for market access were for cancer treatment [19]. As mentioned earlier, REAs of drugs are traditionally based on health outcomes such as overall survival and progression-free survival. However, considering the often-marginal differences in overall survival and progression-free survival for oncological drugs, information on AEs, adherence and quality of life is becoming even more important in REA [20]. Collecting these aspects from RCTs can be difficult, therefore other data sources such as social media may be useful. For the purposes of this explorative review, social media were defined as “a group of Internet-based applications that allow the creation and exchange of user-generated content” [21].

## Methods

An explorative review was performed based on the Preferred Reporting Items for Systematic Reviews and Meta-Analyses guidelines [22]. To identify scientific literature, a search for peer-reviewed published articles was carried out in MEDLINE through the PubMed interface for the period between 1 January 2010 and 28 June 2016. The following search query was used: (Facebook[tiab] OR Twitter[tiab] OR blog[tiab] OR blogging[mesh] OR “social media”[tiab] OR ehealth[tiab] OR e-health[tiab] OR “online community”[tiab] OR “online communities”[tiab] OR “online patient”[tiab] OR “health data”[tiab] OR (online [tiab] AND research[tiab] AND platform*[tiab]) OR (personal*[tiab] AND health[tiab] AND record*[tiab]) OR (online[tiab] AND patient[tiab] AND communit*[tiab]) OR (online[tiab] AND data[tiab] AND shar*[tiab])) AND (oncolog*[tiab] OR cancer[tiab] OR carcinoma[tiab] OR metast*[tiab] OR neoplasms[mesh] OR melanoma[tiab] OR tumor[tiab] OR tumour[tiab]). The reference lists from the literature, which were included based on title and abstract, were hand-searched to identify additional literature. To extend the literature search, the top four health informatics journals according to SCImago Journal and Country Rank [23] were included, namely GigaScience, BMC Medical Research Methodology, Open Bioinformatics Journal, and Journal of Medical Internet Research. The websites of these health informatics journals were hand-searched by assessing theme issues and by using the following keywords: “oncology, cancer, carcinoma, metastasis, neoplasm, tumor, tumour, blog, blogging, social media, e-health, online or health data”.

A Google search was conducted in July and August 2016 to identify grey literature, such as relevant websites, by combining the following keywords: “social media”, “online patient”, “online research platform”, “relative effectiveness”, “health research”, “effectiveness research”, “pharmacovigilance”, “adherence”, and “to measure quality of life”. Before each search, the history of the browser was cleared to ensure findings would not be influenced by previous search queries. Due to the vast number of websites retrieved through the Google search, only websites that collect health data online, focus on patient-reported outcomes, or provide online information on drugs and conditions were deemed relevant for further analysis. The selection of relevant websites was also based on consensus between the authors RK and RtH. These websites were hand-searched to identify grey literature by browsing through the website in search of relevant reports or documents and by using the following keywords: “social media”, “internet”, “Facebook”, “Twitter”, “pharmacovigilance” or “health research”. These keywords were different from those used for the Google search due to the character of the platform (ie, a Google search is inherently different from searching a website). The following websites were included: PatientsLikeMe, Microsoft HealthVault, Dossio, CureTogether, WhatNext, MyGly, Drug Information Association, WEB-RADR, National Patient-Centered Clinical Research Network, College ter Beoordeling van Geneesmiddelen, Handle My Health, European Alliance for Personalized Medicine, Lareb, WHO Monitoring Centre for Pharmacovigilance Uppsala, PEW Research Center, Social Media Research Foundation, Treato, MediGuard, Healthy.me, and iVitality.

The review was conducted by four reviewers (RK, AM, RtH and KM) and the resulting literature was independently screened by the reviewers for eligibility. The titles and abstracts from scientific literature were assessed by RK, AM and KM, while grey literature was assessed by RK and RtH. Literature was considered eligible for inclusion when it was: 1) published between 1 January 2010 and 28 June 2016, 2) available in English, 3) examples were provided where social media were used to collect health data, 4) literature focused on cancer or cancer treatment, and 5) literature was either a peer-reviewed original research article or a report that was available in the public domain. We excluded literature that did not meet all inclusion criteria. Relevant full articles and reports were retrieved and reviewed for inclusion.

Two reviewers (RK and AM) independently extracted data from all included articles and reports using a predefined data abstraction form. Information on study characteristics (eg, study design, study period, type of social media used), and the strengths, limitations and acceptability of using social media to generate health data were extracted. Disagreements in data extracted were resolved by consensus amongst RK and AM.

A descriptive qualitative analysis of the extracted data was carried out, since the topics, methods and outcomes of included literature were notably diverse.

## Results

**Figure 1:**
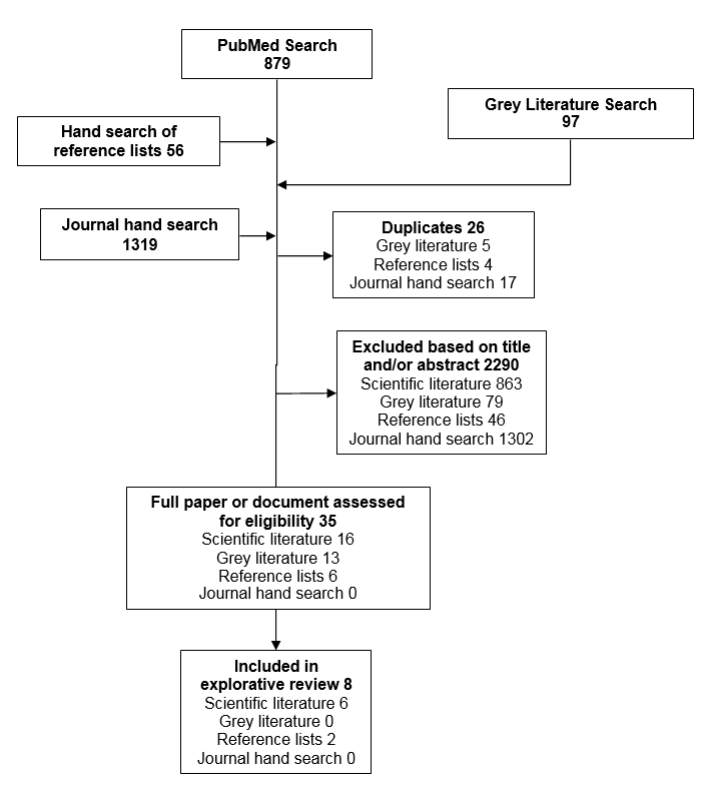
Flowchart of the literature review process.

A total of 2351 citations were identified from scientific literature (n=879), a hand search of reference lists from scientific literature (n=56), grey literature (n=97), and a hand search of health informatics journals (n=1319). From these, a total of 2290 citations were excluded based on title or abstract, additionally 26 duplicates were excluded. Of the 35 full scientific publications and documents assessed, 27 were excluded: 15 citations did not provide an example of health data collection, 9 were not oncology-specific, and 3 provided insufficient information on the collection of health data. Data were abstracted from a total of 8 scientific publications (Figure 1).

Table 1 provides an overview of the eight scientific publications included. Different types of cancer and medications were assessed in each of the publications. The focus of all eight articles was testing the feasibility and added value of generating health data from social media, such as AEs, QoL, adherence, symptom occurrence and experience from social media.

Table 2 shows that publications differed substantially in study design, study period, the number of posts analyzed and the number of respondents included in the analysis. Forum topics and discussions were assessed in four papers, in two studies a survey was posted on the Facebook page of either a patient community or support group, in one study Twitter conversations were assessed and in one study an online patient platform was used to disseminate a survey. Of the eight studies, a total of four studies collected health data on AEs [24,25,28,30]. More specifically, three of these publications presented the AEs identified on the forums included [24,28,30], while the fourth publication focused on comparing AEs mentioned online to AEs reported to the FDA [25]. Another three studies collected health data on quality of life (QoL) [26,27,31]. Each study used different QoL instruments, such as the Concerns About Recurrence Scale scores [31], and short form-36 health survey [26]. Finally, one study focused on identifying symptom (co-) occurrence [29]. In addition to the main outcome measures, van der Heijden et al, McCarrier et al, and Zaid et al [26,27,31] collected data on socio-demographic factors and disease specific characteristics. Furthermore, Beusterien et al collected health data on physical functioning and emotional impacts [24], and Mao et al collected information on adherence by mapping decisions about continuing or stopping treatment [28].

The four publications that used forums to collect health data varied substantially in the explanation for their forum selection (Table 3). For example, Beusterien et al used two search engines and two different computers for their forum search which they repeated every other day for two weeks. Additionally, they used selection criteria to include the two forums (ie, site active >5 years, >12,000 posts on forum, >20 individuals currently browsing, and >10 new posts per day) [24]. Meanwhile, Marshall et al selected one forum without clarifying selection criteria for the selected forum [29]. The other four publications, making use of Twitter, Facebook or an online patient platform, selected this social media platform due to the access of a large volume of health data [25] or access to a patient community [26,27,31].

Regarding the use of automated processes to collect health data from social media, two publications specifically indicated to have used a web crawler [28,29] and one publication made use of the Twitter application programming interface [25]. Two of the included publications indicated to have collected all the forum posts related to search terms without specifically indicating the collection method used [24,30] and three publications used the social media platform to distribute a survey [26,27,31]. Automated techniques were used by Freifeld et al, Mao et al and Marshall et al to analyze the health data collected [25,28,29]. Freifeld et al used a tree-based dictionary-matching algorithm to identify specific text from the forum posts collected, and furthermore used a Natural Language Processing (NLP) semi-automated classifier was used to identify AEs [25]. Mao et al also used NLP to identify AEs [28], and Marshall et al used NLP in a data mining algorithm to identify symptoms [29]. The remaining five publications made use of content analysis [24,27], descriptive or quantitative analysis (eg, chi-squared test) [26,31], or labelled forum posts manually [30].

**Table 1:** Overview of included scientific publications.

Study	Aim	Cancer Type	Drug
Beusterien et al 2013 [24]	To better understand patient experience with colorectal cancer chemotherapies in the real-world setting	Colorectal cancer	Chemo-therapeutic agents
Freifeld et al 2014 [25]	To evaluate the level of concordance between Twitter posts mentioning AE^a^-like reactions and spontaneous reports received by a regulatory agency	N/A^b^	Methotrexate^c^
van der Heijden et al 2016 [26]	To investigate whether we could use crowdsourcing via Facebook and online surveys for medical research purposes on pigmented villonodular synovitis	Pigmented villonodular synovitis	N/A
McCarrier et al 2016 [27]	To explore the feasibility of using social media-based patient networks to gather qualitative data on patient-reported outcome concepts relevant to chronic lymphocytic leukaemia	Chronic lymphocytic leukaemia	N/A
Mao et al, 2013 [28]	To understand frequency and content of AE’s and associated adherence behaviors discussed by breast cancer patients related to using aromatase inhibitors	Breast Cancer	Aromatase inhibitors
Marshall et al, 2015 [29]	To identify and examine symptom patterns generated by data extracted from a breast cancer forum, and compare these findings to an analysis of symptoms reported by breast cancer survivors enrolled in a research study and who responded to a symptom checklist	Breast Cancer	N/A
Pages et al, 2014 [30]	To describe the characteristics of AE’s reported by patients exposed to oral antineoplastic agents in an online discussion, and compare these with those reported by health professionals as recorded in the French pharmacovigilance database	Cancer	Oral antineoplastic agents
Zaid et al, 2014 [31]	To determine the feasibility of using social media to perform cross-sectional epidemiologic and quality of life research on patients with rare gynaecologic tumours	Neuroendocrine carcinoma of the cervix	N/A

^a^AE: adverse events.

^b^N/A: not applicable.

^c^This study assessed adverse events reported in social media for a total of 23 drugs and 4 vaccines, including 1 drug (methotrexate) specific for oncology.

In Table 4 the strengths and limitations of health data generated through social media that were identified in the eight included publications are presented. Five publications identified the ability to assess patient perspectives as an important strength [24,25,28-30]. The ability to access patients who have rare diseases or are distributed over wide geographic areas was considered a major strength by five publications [26-29,31]. Furthermore, Freifeld et al, Marshall et al and Pages et al emphasized that social media should complement conventional (pharmacovigilance) methods, since a difference between results from social media and conventional methods may be present [25,29,30]. For example, patients were shown to report different AEs compared to health professionals who traditionally provide this information [30]. Other strengths identified included the efficient collection of patient-reported outcomes [24], the short time-period needed to survey patients [29,31], and the identification of new or unlabelled AEs [30].

Limitations of social media-generated health data mainly focused on validating authenticity, selection bias, information bias, and the inability to actively probe patients for responses. Validating authenticity focuses on the difficulty of verifying the accuracy of information provided through social media [26,29], such as verifying whether posters have the disease [27,31] or are indeed on the drugs [24,27] they discuss. Regarding selection bias, publications reported differences in the patient population that use social media compared to those who do not; for example, patients using social media are conventionally more highly educated [24,29], are more likely to be female [26,27], may have a different symptom experience [28], and are generally younger [27,29,31]. With regards to information bias, Freifeld et al and Pages et al reported duplication of posts [25,30], Mao et al reported multiple posts by the same patients [28], and Freifeld et al indicated that patients may not identify AEs correctly [25]. Finally, several publications mentioned the inability of using social media to actively probe patients for responses [24,27,29]. For example, patients may use alternative wording than that which researchers anticipate, which could lead to misclassifying symptom experiences [29].

Regarding the acceptability of using social media to generate health data, Pages et al indicated that pharmaceutical companies are already using this type of data to gather information on AEs from patient perspectives [30]. Furthermore, Beusterien et al indicated that in patient-reported outcomes research, patient perspectives are commonly accepted with regards to disease and treatment impact [24], and both Freifeld et al and van der Heijden et al noted the importance of insights into the patient perspective provided by social media research for regulatory authorities [25,26]. However, Freifeld et al was also cautious on the use of social media to generate health data [25]. Reasons for their caution was the need to still establish its role in pharmacovigilance as social media are not yet used in routine surveillance. Additionally, they indicated that data acquisition from social media and automation need to be improved.

**Table 2:** Study characteristics of included scientific publications that use social media to collect health data.

Study	Study design	Study period	Posts analysed	Respondents	Type of social media used to collect health data	Type of health data collected
Beusterien et al 2013 [24]	Cross-sectional	52 days	1522	264	2 disease-specific forums	Adverse events, physical functioning & emotional impacts
Freifeld et al 2014 [25]	Retrospective	7 months	6,900,000	N/A^a^	Twitter	Adverse events
van der Heijden et al 2016 [26]	Prospective	70 months	N/A	272	Facebook (patient community)	Socio-demographic factors, disease-specific characteristics^b^, functional outcome, and QoL^c^
McCarrier et al 2016 [27]	Cross-sectional	4 months	N/A	50	Online patient platform	Socio-demographic factors, disease-specific characteristics^d^, experience of symptoms, perceptions about treatment, and QoL
Mao et al 2013 [28]	Retrospective	8 years	1,235,400	N/A	12 disease-specific forums	Adverse events and adherence
Marshall et al 2015 [29]	Retrospective	8 years	50,426	12,991	1 disease-specific forum	Symptom occurrence, co-occurrence, and similarity index of 25 preselected symptoms.
Pages et al 2014 [30]	Retrospective	1 year	111	66	5 health forums	Adverse events
Zaid et al 2014 [31]	Cross-sectional	30 days	N/A	57	Facebook (support group)	Socio-demographic factors, disease-specific characteristics^e^, and QoL

^a^N/A: not applicable.

^b^Disease-specific characteristics include clinical presentation, findings on imaging and biopsy material, type and localization of disease, surgical and adjuvant treatment, local recurrences, and post-operative complications.

^c^QoL: quality of life.

^d^Disease-specific characteristics include self-reported current chronic lymphocytic leukaemia stage, performance status, and past and current treatment.

^e^Disease-specific characteristics include clinical presentation, initial work-up, treatments, past and current disease status, follow-up, and recurrence pattern.

**Table 3:** Selection of social media platform and use of automated techniques by included literature that use social media to collect health data.

Study	Clear explanation for selection of social media platform	Web crawler used for collecting social media health data	Automated technique used for analysis of health data
Beusterien et al 2013 [24]	Yes	No	No
Freifeld et al 2014 [25]	Yes	No^a^	Yes
van der Heijden et al 2016 [26]	Yes	No^b^	No
McCarrier et al 2016 [27]	Yes	No^b^	No
Mao et al 2013 [28]	Yes	Yes	Yes
Marshall et al 2015 [29]	No	Yes	Yes
Pages et al 2014 [30]	Yes	No	No
Zaid et al 2014 [31]	Yes	No^b^	No

^a^The Twitter application programming interface (API) was used to identify relevant tweets.

^b^A survey was distributed via the social media platform.

**Table 4:** Strengths and limitations specific to the use of social media to generate health data.

Study	Strengths	Limitations
Beusterien et al 2013 [24]	Patient perspective; efficient and comprehensive collection of PROMS^a^	Validating authenticity: selection bias; no active probing of patient responses; incomplete information of sample
Freifeld et al 2014 [25]	Patient perspective; complementary to pharmacovigilance; rapid information on AEs^b^	Information bias; volume of posts; noisy data
van der Heijden et al 2016 [26]	Access to patients with rare diseases; collection of PROMS; convenient to fill in; long-term follow-up	Validating authenticity; selection bias; low participation rate
McCarrier et al 2016 [27]	Alternative approaches to qualitative data collection; support development of PRO^c^ instruments; access to patients with rare diseases; motivated patients; lower costs per enrolled patient	Validating authenticity; selection bias; no active probing of patient responses; not achieving concept saturation; larger sample sizes needed
Mao et al 2013 [28]	Patient perspective; access to patients distributed over wide geographic areas; increased generalizability due to more diverse patient population; observed frequency key AEs reflected those reported in traditional studies	Selection bias; information bias; frequency data is not an indication of prevalence AEs
Marshall et al 2016 [29]	Vast quantities of data; easily accessible information; short time-period; access to patients with rare diseases; low costs; patient perspective; complementary to traditional studies	Validating authenticity; selection bias; noisy data; no active probing of patient responses; incomplete information of sample; data quality or format inadequate; ethical considerations; misinterpretation of posts
Pages et al 2014 [30]	Patient perspective; complementary to pharmacovigilance; identification new or unlabelled AEs	Information bias
Zaid et al 2014 [31]	Access to patients with rare diseases and that are distributed over wide geographic areas; short time-period; motivated patients	Validating authenticity; selection bias

^a^PROMS: patient-reported outcome measures.

^b^AE: adverse event.

^c^PRO: patient-reported outcome.

## Discussion

This explorative review demonstrates that, within the field of oncology, social media could be used for assessing AEs by collecting health data from forums and to evaluate QoL through Facebook or online patient platforms. Social media provides an opportunity to efficiently assess patient perspectives and collect health data from patients with rare diseases that are distributed over wide geographic areas. However, validating the authenticity of health data from social media is difficult, and is prone to selection and information bias. Furthermore, this type of data should be used complementary to traditional forms of research. Finally, this review provides additional insights, compared to reviews that focus on social media to inform pharmacovigilance [32,33], by focusing on the use of social media to inform relative effectiveness assessments.

Arguably, the results found in this review on social media-generated data in oncology may not be generalizable to other fields of medicine, since different types of health data, social media or analysis may be of importance in other fields of medicine. However, many studies conducted in fields of medicine other than oncology similarly focused on identifying AEs [32-38], suggesting our results are at least partially generalizable. Although little is known about assessing QoL through social media in other fields of medicine, there is potential for this mode of health data collection since QoL is often difficult to measure in RCTs and observational studies [20]. Finally, as our results show, another aspect of relative effectiveness that may be assessed through social media is treatment-switching and adherence behavior. A few pharmaceutical companies have been assessing this aspect already, thus demonstrating its potential [14,15,39]. Given the possibility of social media to generate data on AEs, QoL, and treatment-switching and adherence behavior, there is a great potential for social media-generated health data to enrich REA by incorporating information on these aspects.

One caveat of using social media to collect health data that requires special attention is the lack of clear methodological guidance. Standardized approaches to collecting health data from social media are necessary to ensure comparability and reproducibility between studies. For example, posts may either be extracted manually or by automated processes. The interpretation of these posts could also be done manually or by automated processes. However, some argue that automated processes may be unable to successfully interpret sarcasm in text posted on social media [25], while others argue that automated natural language processing could assist in analyzing the vast amounts of data available on social media [33,40,41]. Another methodological issue involves the use of correct search terms, as posts may include misspellings, non-medical terms, and slang [25,33,42]. Additionally, several studies reported important methodological limitations to consider when assessing data from social media, which include validating authenticity (eg, posts may be not genuine) [43-45], selection bias (eg, social media users may differ in age, gender, ethnicity and physical location compared to non-users) [42,44,45] and information bias (eg, patients may be taking a specific drug but fail to report the drug or its effects) [43,45]. To manage these methodological limitations, it is important to systematically assess the risk of bias to determine the quality of the health data collected through social media. Extracting relevant health data from social media may be difficult and challenging due to the issues described above. Clear and uniform methodological guidance may improve the extraction, interpretation and subsequent use of social media to collect health data. An additional caveat that may hamper the use of social media for collecting health data for REA is the perceived risk of easy manipulation. A recent example of manipulation in social media was the circulation of fake news on social media during the 2016 elections in the United States of America [46-48]. These kind of examples affects the ability of social media users to discern what is true and correct information. However, although manipulation may occur, many still use social media to find information and to exchange experiences. Therefore, harnessing and analysing the vast amount of health data available on social media remains important.

Although caveats can be recognized in the use of social media-generated health data, the added value of collecting information on patients’ perspectives and experiences towards relative effectiveness (eg, AEs, quality of life, switching-behavior) should be highlighted. For example, health data collected through social media may uncover AEs that occur after long-term use of new drugs, or they may detect AEs earlier compared to traditional methods [44,49], or provide insights that are not available in published literature (eg, diabetes patient experiences with laser therapy) [12]. Additionally, social media may be a better source to identify AEs that are mild or symptom-related compared to more traditional methods [44]. However, health data collected through social media should be used in conjunction with traditional methods to ensure the collection of a comprehensive overview of aspects that can provide information for REA.

Important for the comprehensiveness of this review is that we assessed both academic and grey literature, which minimizes the possibility of missing important insights. Additionally, we ensured the quality of the review through data abstraction conducted by two authors, which allowed a better substantiation of deductions made.

One limitation of this review was the focus on oncology, which may have resulted in missing literature on other aspects related to REA that could potentially be collected using social media. For example, PatientsLikeMe, an online patient platform that allows patients to share health data or exchange experiences on conditions and medications, published a few studies on the effectiveness of off-label drug use [43,50]. Additionally, PatientsLikeMe published a study focused on assessing the impact of menopause on disease severity in patients with multiple sclerosis. [51] These types of data may contribute to providing information for REA. The focus on oncology in this review was deemed appropriate since many new drugs are developed in the field of oncology, studies that assess these new drugs can be small and incomplete, and the European Medicines Agency and the European Network for Health Technology Assessment are also putting focus on the assessment of oncological drugs.

A second limitation relates to the search strategy employed in this explorative review. Firstly, the broad definition of social media that was used in this review may not allow for differentiating between passively collecting data (eg, by collecting posts from a forum) and actively collecting data (eg, by posting a survey on Facebook). There may be a difference in the information available from passively collecting information that patients discuss and post on social media, compared to actively posing questions to these patients in a survey. Secondly, by employing one database for our scientific and grey literature search we may have missed studies published in relevant journals that are not indexed by PubMed or grey literature that was not identified by the Google search engine. To overcome this limitation to some extent, we hand-searched the reference lists of included studies, based on title and abstract, and identified a few articles that had not been captured in the PubMed and Google search.

Social media may be a potential source of RWD for REA, particularly on aspects such as AEs, occurrence of disease-specific symptoms, adherence behavior, and QoL. This potential has not yet been fully realized due to methodological limitations that accompany social media-generated health data, like information bias and selection bias, as well as the limited acceptability of such data. However, the degree of usefulness of such data for relative effectiveness assessment should be further explored. Moreover, methodological guidelines and tools should be developed to address the limitations mentioned above.

## Abbreviations

HTA: health technology assessment
NLP: Natural Language Processing
QoL: quality of life
RCT: randomized controlled trial
REA: relative effectiveness assessment
RWD: real-world data
